# A Full-Spectrum Evaluation of Sigma-1 Receptor (S1R) Positron Emission Tomography (PET) Radioligands from Binding Affinity to Clinical Imaging

**DOI:** 10.3390/molecules30214296

**Published:** 2025-11-05

**Authors:** Francesco Mastropasqua, Friedrich-Alexander Ludwig, Carmen Abate

**Affiliations:** 1 Department of Pharmacy-Pharmaceutical Sciences, University of Bari Aldo Moro, Via E. Orabona, 4, 70125 Bari, Italy; 2 Department of Experimental Neurooncological Radiopharmacy, Institute of Radiopharmaceutical Cancer Research, Helmholtz-Zentrum Dresden-Rossendorf (HZDR), Research Site Leipzig, Permoserstraße 15, 04318 Leipzig, Germany

**Keywords:** sigma-1 receptor (S1R), positron emission tomography (PET), radioligands

## Abstract

Several pieces of evidence have demonstrated the sigma-1 receptor (S1R) as a druggable protein with important therapeutic potentials, including neurodegeneration, cancer, and neuropathic pain. The density of S1R is altered in pathological processes so that its imaging is under study for diagnostic purposes. Thus, research has been focused on the development of S1R positron emission tomography (PET) radioligands, not only as diagnostic tools but also as powerful means to assist in the drug-development process. Herein, we comprehensively review the most important S1R PET radiotracers belonging to different classes that have been developed in the last two decades. Starting from the structural modifications impacting on the S1R affinity and selectivity, we report (i) the differences in metabolism and pharmacokinetics, (ii) the in vivo behavior in different animal models, (iii) the in vitro autoradiography outcomes, and (iv) the dosimetric profiles. The successful use of the best-performing S1R PET radiotracers in the characterization of novel S1R drugs is also reported together with the approaches to assess the potential for clinical translation. What emerges from this review is that, although the development of reliable PET agents appears to be extremely challenging, these radiotracers hold incredible potential and play a fundamental role in the exploitation of S1R in health and disease.

## 1. Introduction

Sigma receptors (SRs) are classified into two subtypes: sigma-1 receptor (S1R) and sigma-2 receptor (S2R, also known as transmembrane protein 97, TMEM97). These subtypes differ in molecular weight, tissue distribution, and drug-binding selectivity [1]. Notably, the primary amino acid sequences of the two proteins show no similarity; their crystal structures reveal a completely different folding, but the two binding sites are structurally convergent [2,3,4,5]. S2R is a four-transmembrane ER protein that plays a crucial role in cell proliferation and apoptosis as well as in neurodegeneration [6]. S1R is a small transmembrane protein (~25 kDa) predominantly located in the endoplasmic reticulum (ER), particularly enriched at mitochondrion-associated membranes (MAMs), regions of the ER in close contact with mitochondria [7,8]. The gene encoding S1R is located on chromosome 9 at band p13 (in both humans and mice) and consists of four exons and three introns [9,10]. Under physiological conditions, S1R forms calcium-sensitive complexes with the ER chaperone BiP (Binding Immunoglobulin Protein) [1,11]. Upon activation by specific ligands, S1R dissociates from BiP and translocates to various cellular compartments, including mitochondria, the nucleus, and the plasma membrane. In these locations, S1R performs essential functions such as stabilizing the inositol 1,4,5-trisphosphate receptor (IP_3_R), facilitating calcium transfer from the ER to mitochondria, and supporting ATP production [12,13]. S1R is expressed in both the central nervous system (CNS) and peripheral tissues. Within the brain, where it is widely distributed, it is particularly abundant in areas involved in memory processing, emotional regulation, and sensorimotor functions [2,14]. S1R plays a neuroprotective role and is implicated in the pathophysiology of several neurological disorders, including Parkinson’s disease (PD) [15], depression [16], Alzheimer’s disease (AD) [17,18], pain [19,20], and cancer [21,22,23]. More recently, S1R has also been proposed as a potential therapeutic target for COVID-19 [24,25]. Importantly, two S1R ligands, such as Blarcamesine and Pridopidine, are in advanced clinical phases for the treatment of AD and Huntington’s Disease (HD), respectively, and their use in related pathologies is also under investigation [26,27,28]. Over the years, positron emission tomography (PET) has become an invaluable tool for advancing our understanding of S1R’s role in various diseases [29,30], such as cancer [31] and AD [32]. PET is a non-invasive nuclear imaging technique that provides biochemical information about tissues, overcoming the anatomical limitations of conventional imaging methods [33,34,35]. The integration of PET with computed tomography (CT) or magnetic resonance imaging (MRI) in multimodal systems (e.g., PET-CT and PET-MRI) enables simultaneous acquisition of functional and anatomical data [36]. The most commonly used radionuclides for PET imaging of S1R are fluorine-18 (^18^F) and carbon-11 (^11^C). As the half-life of carbon-11 is very short (20.4 min), its clinical use is limited because each step needs to be performed very rapidly, including radiochemical synthesis, purification, final formulation, and dispensing for intravenous injection to a living subject. [37]. In contrast, ^18^F has a longer half-life (109.8 min), allowing for more extended synthesis protocols and whole-body imaging [38,39]. Several reviews have been published on the role of the S1R in PET imaging. So far, they have focused on fluorinated tracers, with special attention to [^18^F]Fluspidine and its enantiomers [40,41], application in tumors [42] or AD [43]. The present review, instead, provides a comprehensive overview of the S1R PET radiotracers developed since the early 2000s, comparing these tracers across key evaluation parameters, including binding affinity and selectivity, radiochemical synthesis, autoradiography, metabolism, kinetic modeling, dosimetry, in vivo behavior in animal models of different pathologies, and translation into clinic.

## 2. Development of High Affinity and Selective S1R Radioligands

**Figure 1:**
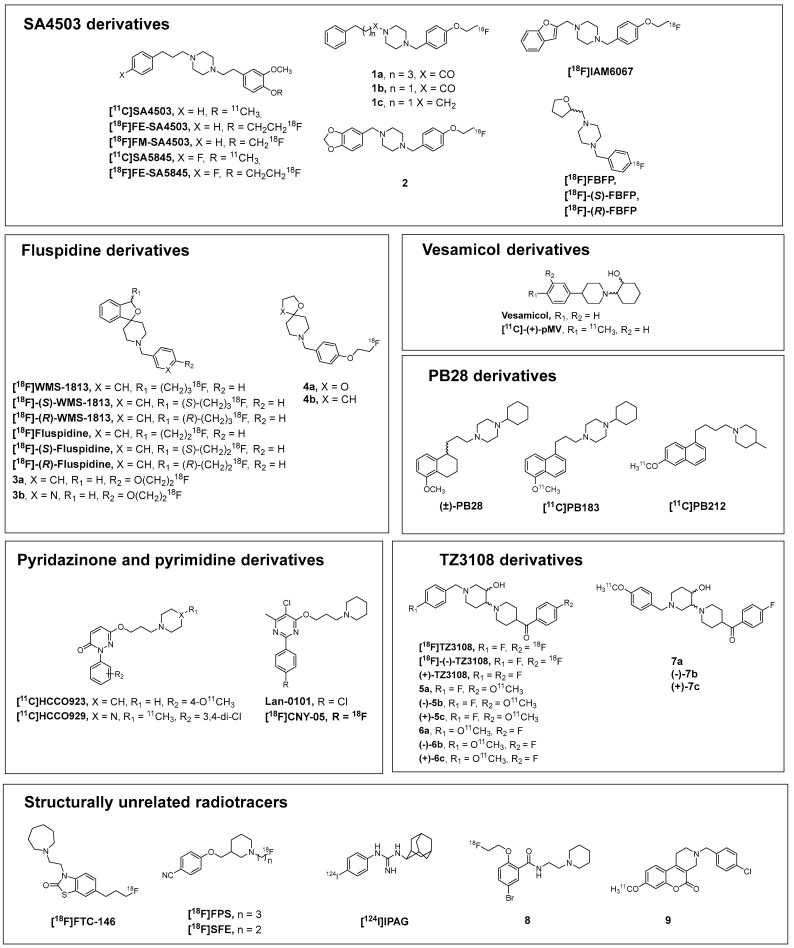
Structures of known PET tracers and their lead compounds developed for S1R imaging.

In this section, we examine the development of the main PET radiotracers for S1R identified to date, comparing how their *K_i_* values vary according to structural modifications. The compounds are grouped based on their lead compounds (Figure 1), and their affinity data for S1R and S2R are reported. Additionally, the corresponding lipophilicity values (logP/logD_7.4_) are reported (Table 1), since for brain PET radiotracers, the ability for passive entry into the brain is optimal with logD_7.4_ values in the range of 2.0–3.5 [44].

### 2.1. SA4503-Derivatives

**Figure 2:**
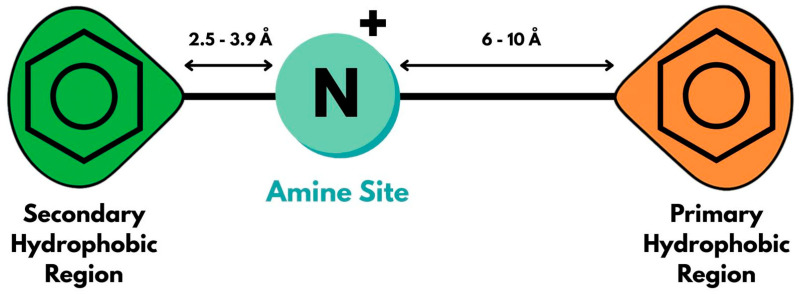
Schematic representation of the “Glennon pharmacophore”, highlighting the key structural features common to S1R ligands. Adapted from Ref. [48].

[^11^C]SA4503 is one of the first PET radiotracers developed for the S1R, which quickly became the benchmark for subsequent radiotracer development. Originally synthesized as a potent and selective S1R agonist [45], SA4503 was adopted for PET imaging only after its 3,4-dichloro analog, [^11^C]SA6298, was found to be unsuitable for in vivo brain imaging due to high nonspecific binding [46]. [^11^C]SA4503 demonstrated strong potential for mapping S1R both in the brain and in peripheral organs [47]. SA4503, like the other PET radiotracers discussed in this review, aligns with the “Glennon pharmacophore” model (Figure 2). Its structure features a central positively charged nitrogen atom connected to two hydrophobic or aromatic moieties, located approximately 6–10 Å and 2.5–3.9 Å away from the nitrogen, respectively [48].

[^18^F]FE-SA4503, [^18^F]FM-SA4503, [^11^C]SA5845, and [^18^F]FE-SA5845 were derived from structural modifications of one or both aromatic rings of SA4503. While [^18^F]FE-SA4503 and [^18^F]FM-SA4503 retained affinity and selectivity profiles comparable to the parent compound, the introduction of an additional fluorine atom on the second aromatic ring in [^11^C]SA5845 and [^18^F]FE-SA5845 resulted in a marked reduction in selectivity [49,50,51]. Based on the SA4503 structure, a lot of compounds have been synthesized. Compounds **1b** and **1c** are structural variants of SA4503, differing primarily in the length of the linker between the piperazine nitrogen and the “second hydrophobic region”, which was reduced from two to one carbon atom. Additionally, the substituents on the same aromatic ring were changed from a substituted 3,4-dimethoxy group to a substituted 4-fluoroethoxy group. Both modifications resulted in improved affinity and selectivity. In contrast, compound **1a** features alterations in the linker in the opposite part of the molecule, extending the chain from three to five carbon atoms, and more significantly, the basic nitrogen of the piperazine was transformed into an amide group. This compound also demonstrated enhanced affinity and selectivity [52]. Similar modifications were made to obtain compound **2**. The second hydrophobic region and the linker connecting it to the piperazine were modified in the same way as in compounds **1a–c**; what differs is the first hydrophobic region. A dioxolane ring was fused to the aromatic ring, and the linker was shortened to a single carbon atom. The result was an affinity for S1R roughly comparable to that of SA4503 and compounds **1a–c**, with selectivity remaining greater than 100-fold [53]. To obtain [^18^F]IAM6067, only the primary hydrophobic region was modified, this time represented by a benzofuran linked to a piperazine via a methylene group. The affinity toward S1R remained within the same range as for the previously mentioned compounds, while the selectivity was over 100-fold [54]. The fluoroethoxy group was replaced with a fluorine, and the benzofuran ring was simplified in compound [^18^F]FBFP in a tetrahydrofuran one. Compared to [^18^F]IAM6067, the S1R affinity value of [^18^F]FBFP remained virtually unchanged, whereas selectivity against S2R was reduced [55].

### 2.2. Fluspidine Derivatives

The initial molecules of the spirocyclic series synthesized shared the same core structure as the later developed [^18^F]WMS-1813 and [^18^F]Fluspidine but had different substituents on the oxolane ring (R_1_ = H, OCH_3_, and CN, Figure 1). Despite exhibiting sub-nanomolar affinity for S1R and high overall binding affinity, these compounds were found to be metabolically unstable [56]. Through a series of structural modifications targeting the nitrogen-bound hydrophobic region of the piperidine ring and the benzyl-linked side chain, [^18^F]WMS-1813 emerged as a promising candidate [57]. A more detailed structure–activity relationship (SAR) study later led to the development of [^18^F]Fluspidine, the lower homolog of [^18^F]WMS-1813, in which the side chain was shortened from a propyl to an ethyl chain. [^18^F]WMS-1813 and [^18^F]Fluspidine exhibited comparable affinity and selectivity profiles [58]. Since the S1R is known to exhibit stereoselectivity [59], enantiomeric resolution was performed for both compounds. In the case of [^18^F]WMS-1813 and [^18^F]Fluspidine, the two enantiomers displayed comparable affinity and selectivity toward S1R. However, in both cases, the *S*-enantiomer demonstrated higher metabolic stability and more favorable pharmacokinetic properties [60,61]. Starting from [^18^F]Fluspidine, numerous compounds featuring a spirocyclic scaffold have been synthesized. Compound **3a** is a direct derivative of Fluspidine, in which the dihydroisobenzofuran-linked ethyl chain has been removed and a fluoroethoxy group was introduced at the para position of the (non-fused) benzene ring. The S1R affinity of this last compound was comparable to that of Fluspidine, but with significantly lower selectivity against S2R (Table 1) [62]. Compound **3b**, which is an isostere of compound **3a**, by contrast, showed reduced affinity and selectivity [63]. Compound **4a** represents a structural simplification of compound **3a**, in which the isobenzofuran moiety has been replaced by a 1,3-dioxolane one. This modification, introduced to decrease the logD_7.4_ value for peripheral tumor imaging [64], also resulted in a decreased affinity and selectivity. Compound **4b**, an isoster of **4a** in which the 1,3-dioxolane at the spiro position was replaced by a tetrahydrofuran one, showed a further reduction in both the S1R affinity and selectivity [65].

### 2.3. Vesamicol Derivatives

Another category of compounds consists of vesamicol derivatives. Vesamicol, which inhibits acetylcholine transport, also exhibits moderate affinity for the S1R and poor selectivity. However, introducing a methyl group at the para position of the aromatic ring significantly improved S1R affinity and selectivity ([^11^C]-(+)-pMV) [66]. Additional structural modifications more focused on non-PET emitter radionuclides were introduced in vesamicol derivatives, which will not be treated in this review.

### 2.4. PB28-Derivatives

[^11^C]PB183, on the other hand, is derived from the pan-sigma receptors subnanomolar affinity PB28 and features a naphthalene core in place of the tetrahydronaphthalene scaffold [67]. This compound with similar subnanomolar S2R affinity displayed a 1-digit S1R affinity [68] in prostatic cancer cell homogenates. These properties, together with the activity at the P-gp efflux pump (PB183 is transported by P-gp), suggested it as a potential PET radioligand to target peripheral solid tumors. However, no further studies were conducted on this compound. In [^11^C]PB212, which also bears a naphthalene core, the linker was extended by one carbon atom, and the cyclohexylpiperazine moiety was replaced with a 4-methylpiperidine one in order to improve the S1R selectivity. These structural modifications lead to a picomolar S1R affinity and a 2-digit nanomolar S2R affinity, thus importantly improving the S1R selectivity, prompting the study as an S1R PET diagnostic, and revealing the potential of [^11^C]PB212 for the imaging of peripheral S1R [69].

### 2.5. Pyridazinone and Pyrimidine Derivatives

[^11^C]HCCO923 and [^11^C]HCCO929 belong to the pyridazinone class. The replacement of two Cl atoms in [^11^C]HCCO923 with a methoxy group and the substitution of the 4-methylpiperazine with a piperidine led to [^11^C]HCCO929 with a 2-fold higher S1R affinity and S2R/S1R selectivity than [^11^C]HCCO923 [70]. A compound structurally related to [^11^C]HCCO929 is [^18^F]CNY-05, which features a pyrimidine core instead of a pyridazinone. [^18^F]CNY-05 was developed from Lan-0101 [71,72] by replacing the Cl with a F atom. This substitution led to an extremely high affinity for S1R but to a 100-fold lower S1R selectivity against S2R [72].

### 2.6. TZ3108-Derivatives

[^18^F]TZ3108 with an S1R subnanomolar affinity and >3000-fold selectivity towards S2R derives from an SAR study, during which several structural modifications were made to the two aromatic rings [73]. The enantiomers of TZ3108 were separated, revealing (+)-TZ3108 as the eutomer. However, despite the exceptionally high selectivity of the eutomer, the (–)-enantiomer showed faster clearance, making it overall more favorable than both the (+)-enantiomer and the racemate. Of the two enantiomers, only the (–)-isomer was radiolabeled with ^18^F [74]. Starting from TZ3108, a new series of ^11^C-derivatives with small structural changes was produced. Replacement of the F-atom with an OCH_3_ group at position R_2_ (compounds **5a–c**) resulted in reduced S1R affinity and, more notably, decreased selectivity. On the other hand, the same replacement at position R_1_ (compounds **6a–c**), while leading to lower affinity than TZ3108, improved selectivity. In compounds **7a–c**, the position of the bond between the two piperidine rings was modified, in addition to the F/OCH_3_ substitution at R_1_, resulting in reduced affinity and selectivity. In this series as well, the (+)-enantiomer proved to be the eutomer [75].

### 2.7. Structurally Unrelated Radiotracers

[^18^F]FTC146 is the only PET radiotracer for the S1R belonging to the benzothiazolone class, to the best of our knowledge. It is endowed with an S1R picomolar affinity and an exceptionally high selectivity against the S2R, greater than 145,000-fold [76]. This compound originated from an SAR study conducted by Yous S. et al. focused on benzothiazolones as S1R ligands [77].

[^18^F]FPS is one of the first PET radiotracers developed for the S1R, alongside [^11^C]SA4503. Structurally, it belongs to the phenoxymethylpiperidine class and shows subnanomolar S1R affinity [78]. Its lower homolog, [^18^F]SFE, was later developed by shortening the *N*-alkyl chain. However, this modification resulted in a reduction in both S1R affinity and selectivity [79]. [^124^I]IPAG that exhibits both high affinity and selectivity for S1R was developed with the aim of creating a radiolabelable compound incorporating ^124^I and featuring a guanidine moiety as its basic group [80]. In Figure 1, the 2-adamantyl isomer of IPAG has been depicted in line with the structure that has been lately commonly reported. However, the original structure for this S1R antagonist bore the 1-adamantyl radical on the guanidine moiety [81,82].

Compound **8**, belonging to the benzamide class, was designed to exert dual action on both S1R and S2R for the purpose of prostate cancer imaging. Indeed, it displays comparable affinity for both receptors [83].

Compound **9**, which was originally developed for D_4_ receptor imaging, also binds to S1R, a finding that emerged through in vivo studies [84]. No further studies are reported for these last radiotracers.

**Table 1:** Summary table of S1R and S2R binding data and lipophilicity (logD_7.4_/logP).

Tracer	*K*_i_ S1R (nM)	*K*_i_ S2R (nM)	logD_7.4_^#^	References
[^11^C]SA4503	4.6	63.1	2.52 ^#^	[47]
[^18^F]FE-SA4503	8.0	113.2	2.70 ^#^	[49]
[^18^F]FM-SA4503	6.4	250	/	[51]
[^11^C]SA5845	33	9.5	2.60 ^#^	[50]
[^18^F]FE-SA5845	3.10	6.8	2.70 ^#^	[50]
**1a**	4.19	2000	3.56	[52]
**1b**	1.19	2913	2.72	[52]
**1c**	0.31	42.6	3.35	[52]
**2**	1.85	291	1.06	[53]
[^18^F]IAM6067	2.6	486	3.35	[54]
[^18^F]FBFP	3.22	168	0.76	[55]
[^18^F]-(*S*)-FBFP	3.16	126	0.76	[85]
[^18^F]-(*R*)-FBFP	3.23	178	0.76	[85]
[^18^F]WMS1813	1.4	837	3.56	[57]
[^18^F]-(*S*)-WMS1813	0.59	/	3.56	[60]
[^18^F]-(*R*)-WMS1813	1.8	/	3.56	[60]
[^18^F]Fluspidine	0.59	785	2.57	[58]
[^18^F]-(*S*)-Fluspidine	2.3	897	2.57	[61]
[^18^F]-(*R*)-Fluspidine	0.57	1650	2.57	[61]
**3a**	0.79	277	2.41	[62]
**3b**	2.30	327	2.58	[63]
**4a**	5.4	164	0.8	[64]
**4b**	8.62	378	1.6	[65]
[^18^F]FTC-146	0.0025	selectivity > 145,000	1.45	[76]
[^18^F]FPS	0.50	144	2.80	[78]
[^18^F]SFE	5.00	361	2.40	[79]
[^18^F]TZ3108	0.48	1740	2.83	[73]
[^18^F]-(-)-TZ3108	1.80	6960	2.83	[74]
(+)-TZ3108	0.14	2390	2.83	[74]
**5a**	2.5	2790	/	[75]
**(**−**)-5b**	30	3910	/	[75]
**(+)-5c**	0.82	6370	/	[75]
**6a**	2.8	5520	/	[75]
**(**−**)-6b**	4.1	>10,000	/	[75]
**(+)-6c**	0.73	>10,000	/	[75]
**7a**	23.7	502.6	/	[75]
**(**−**)-7b**	24.2	402.6	/	[75]
**(+)-7c**	14.3	408.8	/	[75]
[^11^C]HCCO923	10.3	1146	0.89	[70]
[^11^C]HCCO929	5.6	1528	1.73	[70]
LAN-0101	1.06	1425	/	[71]
[^18^F]CNY-05	0.04	4.72	/	[72]
PB28	0.38	0.68	/	[67]
[^11^C]PB183	0.50	6.50	/	[68]
[^11^C]PB212	0.030	17.9	2.38	[69]
(+)-Vesamicol	31.5	330	/	[66]
[^11^C]-(+)-*p*MV	3.0	40.7	/	[66]
[^124^I]IPAG	9.0	high selectivity *	/	[80]
**8**	6.26	10.2	1.4	[83]
**9**	/	/	3.77 ^#^	[84]

^#^ logP is reported instead of logD_7.4_; “/” Data not reported; * no *K*_i_ published.

## 3. Synthesis of S1R Radiotracers for PET

### 3.1. ^11^C Radiolabeling

Radiochemistry strategies for PET tracers labeled with carbon-11 have evolved considerably over the past two decades. While early methods relied primarily on [^11^C]methylation using [^11^C]CH_3_I or [^11^C]CH_3_OTf, more recent approaches include advanced transformations such as [^11^C]-carbonylation, -cyanation, and -CO_2_ fixation. These methodologies allow the incorporation of the radionuclide into a broad range of functional groups such as amines, alcohols, and thiols under mild conditions, with good radiochemical yields and high molar activities [86,87,88]. Table 2 summarizes the synthetic routes of the ^11^C-labeled compounds along with their corresponding radiochemical yields (RCYs). All the compounds discussed in this review were synthesized using [^11^C]CH_3_I or [^11^C]CH_3_OTf, with variations in the nucleophilic group involved in the methylation reaction. [^11^C]SA4503 and **9** were synthesized by methylation of their respective *O*-desmethyl precursors using [^11^C]CH_3_I in a dimethylformamide (DMF) solution containing NaH (A route). [^11^C]SA4503 was obtained with a decay-corrected RCY of 21–31% and a molar activity (A_m_) between 24 and 76 GBq/μmol. In contrast, **9** showed a considerable RCY of 32–59%, with an A_m_ between 67 and 90 GBq/μmol [47,84]. Similarly, [^11^C]PB212 was prepared by methylation of *O*-desmethyl-PB212 with [^11^C]CH_3_I in DMF, using Cs_2_CO_3_ as the base. The decay-corrected RCY for this reaction is 16–33%, with A_m_ between 39 and 391 GBq/μmol at the end of synthesis [69]. Compounds **5a–c**, **6a–c**, and **7a–c** were all obtained by methylation of their respective *O*-desmethyl precursors using [^11^C]CH_3_OTf in DMF solution containing NaOH (Route B). These compounds were produced with a non–decay-corrected RCY of 16–20%, depending on the individual case. The A_m_ values, however, differed among the compounds: **6c** and **7b** showed the lowest values (54 ± 15 GBq/µmol and 52 ± 9 GBq/µmol, respectively); **5c** and **6b** displayed intermediate values (95 ± 19 GBq/µmol and 90 ± 28 GBq/µmol, respectively); while **5b** and **7c** exhibited the highest values (127 ± 26 GBq/µmol and 110 ± 21 GBq/µmol, respectively) [75]. The synthesis of [^11^C]-(+)-pMV was obtained from the corresponding precursor, (+)-*p*-tributylstannylvesamicol, in the presence of tris(dibenzylideneacetone)dipalladium(0), tri(o-tolyl)phosphine, CuCl, and K_2_CO_3_ in DMF (D route). The RCY is 8–19% and an A_m_ between 41 and 162 GBq/μmol [66]. Both [^11^C]HCC0923 and [^11^C]HCC0929 were synthesized using [^11^C]CH_3_I in DMF, but under different reaction conditions. For the preparation of [^11^C]HCC0923, NaOH and 0.1% trifluoroacetic acid (TFA) in water were used, resulting in a non–decay-corrected RCY of 6–15% and an A_m_ of 48 ± 7 GBq/µmol. In contrast, [^11^C]HCC0929 was obtained using K_2_CO_3_ and 0.1% NH_4_HCOO in water (C route), with an RCY of 3–8% (non–decay-corrected) and an A_m_ of 60 ± 7 GBq/µmol [70].

### 3.2. ^18^F Radiolabeling

Radiolabeling strategies for PET tracers using fluorine-18 have significantly expanded beyond conventional nucleophilic substitution methods such as aromatic nucleophilic substitution (SN_Ar_) or aliphatic displacement reactions [89]. Recent innovations in the synthesis of small molecule radiotracers include copper-mediated radiofluorination of (hetero)aryl boronic acids and esters, deoxy-radiofluorination, the use of spirocyclic iodonium ylide precursors, and [^18^F]fluoromethylation techniques, which enable labeling of a variety of substrates. In general, the clinical implementation of new labeling techniques often creates a need for updated regulations [90,91,92,93,94]. A key consideration is the use of automated systems within shielded containers, which can significantly reduce operator radiation exposure, enhance the reproducibility of radiopharmaceutical preparations, and ensure compliance with radiation protection standards as well as GMP requirements [95]. Table 3 summarizes the synthetic routes of the ^18^F-labeled compounds along with their corresponding RCYs. The most widely used synthetic route involves employing a tosylate group as the leaving group in an SN reaction with [^18^F]fluoride, typically in the presence of Kryptofix 2.2.2 as a phase-transfer catalyst. [^18^F]Fluoride can be introduced as its potassium salt or directly as [^18^F]^−^ in an aqueous K_2_CO_3_ solution (G route). This widely employed method has been used for the synthesis of the compounds [^18^F]WMS-1813, [^18^F]Fluspidine, **3a**, **4b**, **1a–c**, and **2**. In all cases, MeCN was used as the solvent; the main variations concerned the reaction temperature and duration. [^18^F]WMS-1813 and [^18^F]Fluspidine were obtained with decay-corrected RCYs of 35–48% and 35–45%, respectively, and A_m_s of 150–238 GBq/μmol and 150–350 GBq/μmol, respectively [57,96]. Compound **3a** was synthesized with a decay-corrected RCY of 35–60%, and its A_m_ at the end of synthesis ranged from 30 to 55 GBq/μmol [62]. For compound **4b**, the RCY is 12–35% (decay-corrected), with an A_m_ of 94–121 GBq/μmol [65]. Compounds **1a–c** and **2** were obtained with decay-corrected RCYs of 42–55% and 30–50%, respectively, and A_m_s of approximately 120 GBq/μmol and 37 GBq/µmol, respectively [52,53]. For [^18^F]Fluspidine, Maisonial-Besset A. et al. reported an automated synthesis protocol using a module that is faster and more versatile, achieving an RCY of 37 ± 8% [97]. The same route was applied in the synthesis of [^18^F]IAM6067 and [^18^F]FTC146. For [^18^F]IAM6067, the reaction was always conducted in CH_3_CN, yielding a non–decay-corrected RCY of 18% and an A_m_ of 45 GBq/μmol. In the case of [^18^F]FTC-146, the reaction was performed in dimethyl sulfoxide (DMSO) at 150 °C, resulting in an RCY of 4–7% and an A_m_ of 96 ± 44 GBq/µmol [54,76]. The radiochemical synthesis of [^18^F]FTC146 was improved by Sadeghzadeh et al. through the use of a less basic [^18^F]TBAF system in CH_3_CN, resulting in an increased RCY of 41.7 ± 4.4%. This approach provided a more reliable and reproducible route for radiosynthesis [98]. For the compounds [^18^F]FPS and [^18^F]SFE, a similar approach was employed, using Kriptofix 2.2.2, [^18^F]^−^ in an aqueous K_2_CO_3_ solution and MeCN as the solvent but substituting the tosylate leaving group with a mesylate group referred to as the H route. [^18^F]FPS and [^18^F]SFE were obtained with RCYs of 56–70% and 59 ± 8%, respectively. The A_m_s were greater than 74 GBq/μmol for [^18^F]FPS and 107 ± 30 GBq/µmol for [^18^F]SFE [78,79]. Another important approach is the two-step radiosynthesis (E route) involving 2-[^18^F]fluoroethyl tosylate ([^18^F]FETos). The automated synthesis of [^18^F]FETos, developed by Schoultz B.W. et al., enables the production of PET tracers with high chemical purity [99]. This synthetic route was employed for the preparation of [^18^F]FE-SA4503 and compounds **3b**, **4a,** and **8**. For [^18^F]FE-SA4503, the reaction was carried out using NaH in DMF, yielding an RCY of 4–7% and an A_m_ greater than 100 GBq/μmol [49]. DMF was also chosen as the solvent for the synthesis of compound **4a**, with Cs_2_CO_3_ used as the base. The decay-corrected RCY was 15%, and the A_m_ ranged from 25 to 45 GBq/μmol [64]. In the case of compound **3b**, the synthesis was performed using 2 mol/L NaOH in DMSO, resulting in a decay-corrected RCY of 8–10% and an A_m_ between 56 and 78 GBq/μmol [63]. Lastly, compound **8** was synthesized using a solution of tetrabutylammonium hydroxide in CH_3_CN, affording a decay-corrected RCY of 35–50% and an average A_m_ of 226 GBq/μmol [83]. For [^18^F]FM-SA4503, in contrast to [^18^F]FE-SA4503, a two-step radiosynthesis is consistently employed, using bromide as the leaving group instead of tosylate (F route). The RCY is 3–17%, with A_m_ between 39 and 283 GBq/μmol [51]. Regarding [^18^F]TZ3108, it is the only compound for which a _2_ nitro group was selected as the leaving group (I route). The labeling was performed through an aromatic nucleophilic substitution reaction in the presence of Kryptofix 2.2.2 and [^18^F]^−^ in an aqueous K_2_CO_3_ solution, using DMSO as the solvent. This resulted in a decay-corrected RCY of 18–24%, with an A_m_ greater than 74 GBq/μmol [100]. For compound [^18^F]FBFP, radiolabeling was carried out via reductive amination in the presence of NaBH_3_CN and CH_3_COOH, using DMSO as the solvent (M route). The decay-corrected RCY is 20–30%, and the A_m_ ranged from 54 to 86 GBq/μmol [55]. The most innovative approach was employed for [^18^F]CNY-05. In this case, the [^18^F]fluorine was introduced into the aromatic ring via direct substitution of an OH group through an Ru-mediated [^18^F]deoxyfluorination (L route). The decay-corrected RCY is 25–40%, with an A_m_ between 128.5 and 133.2 GBq/μmol [72].

### 3.3. ^124^I Radiolabeling

Unconventional long-lived radioisotopes, such as ^124^I with a distinct half-life, have expanded imaging capabilities. Iodine-124 (t_1_/_2_ = 4.2 days) allows for extended imaging timeframes, making it particularly well-suited to track the slow pharmacokinetics of large biomolecules. Its compatibility with both electrophilic and nucleophilic labeling strategies under mild conditions has established it as a key isotope in immunoPET [101,102].

Table 4 reports the synthetic route of ^124^I-labeled compound along with its corresponding RCY. For [^124^I]IPAG, the radiolabeling was performed using [^124^I]NaI and chloramine-T in ethanol (N route), affording a quantitative RCY and an A_m_ ranging from approximately 0.074 to 6.29 GBq/μmol [80].

## 4. Preclinical Biodistribution and Blocking Studies

In vivo biodistribution and blocking studies are key components of PET tracer validation, providing essential data on pharmacokinetics, uptake, target specificity, and off-target binding. These evaluations are critical to confirm tracer suitability before clinical translation [103]. [^11^C]SA4503 in rat and mice studies, exhibited high initial uptake, followed by a gradual washout over time. Radioactivity in the blood and heart also decreased progressively. Brain uptake was significantly reduced by pre-administration of haloperidol, (+)-pentazocine, or non-labeled SA4503. All three agents showed a substantial blocking effect—up to approximately 80%—indicating a high specific binding to S1R [47,104]. [^18^F]FE-SA4503 exhibited a regional brain distribution in rats similar to that of [^11^C]SA4503. However, it showed a higher initial brain uptake that declined more rapidly, suggesting a lower affinity and more reversible binding to sigma receptors. Additionally, [^18^F]FE-SA4503 demonstrated a gradual increase in bone accumulation of radioactivity over time. Brain uptake was significantly reduced following pretreatment, co-injection, or displacement with haloperidol, as well as by co-injection of SA4503 [49]. [^18^F]FM-SA4503 displayed time–activity curves in the brain and peripheral organs of mice that closely resemble those of [^11^C]SA4503. In the brain, [^18^F]FM-SA4503 uptake increased over the first 30 min before gradually decreasing, whereas [^11^C]SA4503 peaks within the first 5 min and then declines. Specific binding of [^18^F]FM-SA4503, assessed via co-injection with haloperidol, was 68% at 60 min p.i., compared to 60% for [^11^C]SA4503 at 30 min [51]. [^18^F]FE-SA4503, [^18^F]FM-SA4503, and [^11^C]SA4503 exhibited similar regional distributions in the conscious monkey brain, with high uptake observed in cortical areas, thalamus, striatum, and cerebellum—regions known to be rich in sigma receptors. However, their time–activity curves differed significantly. Blocking studies with haloperidol demonstrated a rapid reduction in tracer uptake for all compounds. Notably, [^18^F]FM-SA4503 showed higher specific binding compared to [^11^C]SA4503 (68–83% vs. 50–60% at 60–80 min p.i.) [49,51,105]. [^18^F]FE-SA5845 was evaluated in the conscious monkey brain. Compared to [^18^F]FE-SA4503, it showed lower uptake in the cingulate, temporal, and frontal cortices, but higher uptake in the vermis, thalamus, and occipital cortex [106]. Regarding compounds structurally related to [^11^C]SA4503, compounds **1a–c** exhibited high brain uptake and specific binding comparable to [^11^C]SA4503. Among them, compound **1c** showed the most promising profile; however, its brain kinetics in mice were very slow, which is why this compound was not advanced in nonhuman primates (NHPs) [52]. Compound **2** demonstrated a very high initial brain uptake, surpassing that of both [^11^C]SA4503 and [^18^F]Fluspidine, followed by a slow clearance phase. It also exhibited strong specific binding [53]. In the mouse model, [^18^F]IAM6067 showed rapid brain uptake. Blocking studies with haloperidol resulted in approximately 80% reduction in uptake, and co-injection with CM398 confirmed in vivo selectivity against S2R, demonstrating that [^18^F]IAM6067 is a specific and selective radiotracer [107]. In Papio hamadryas baboons, the tracer displayed high and homogeneous uptake in brain regions known to have high S1R density (cerebral cortex, hippocampus, striatum, amygdala, cerebellum, and brainstem), followed by complete washout. Blocking studies confirmed the results observed in mice [54]. Despite its low logD_7.4_ (0.76), [^18^F]FBFP showed very high initial brain uptake, followed by gradual washout. Blocking studies with SA4503 revealed a reduction in uptake both in the brain and in peripheral organs rich in S1R [55]. Since FBFP possesses a chiral center, enantiomeric resolution was performed, and both enantiomers were evaluated in rodents. Both the *R*- and *S*-enantiomers exhibited high brain uptake and favorable brain-to-blood ratios. Blocking studies with SA4503 resulted in a marked reduction in the uptake of both enantiomers, consistent with the behavior observed for the racemate. In dynamic micro-PET studies, [^18^F]-(*S*)-FBFP demonstrated substantially faster clearance from the brain than the *R*-enantiomer, indicating distinct pharmacokinetic profiles between the two [85]. On the other hand, among compounds with a spirocyclic structure, [^18^F]WMS-1813 exhibited high brain uptake and prolonged retention of radioactivity, indicating favorable brain penetration as well as specific binding. It also showed high initial uptake of radioactivity in most organs known to express S1R. Radioactivity was also observed in the femur, indicating defluorination in vivo. Blocking studies with haloperidol demonstrated a significant reduction in radioactivity uptake in organs with high S1R expression, approximately 80% in the brain, except for the liver [57]. [^18^F]Fluspidine showed higher initial brain uptake (at 5 min) than [^18^F]WMS-1813 and [^11^C]SA4503, and its uptake at 30 min is 40–50% higher compared to its F-propyl derivative and [^11^C]SA4503. Its biodistribution pattern is similar to that of [^18^F]WMS-1813, including radioactivity observed in the femur [96]. Based on [^18^F]Fluspidine, compounds **3a** and **3b** were synthesized. Compound **3a** exhibited better brain uptake than both [^11^C]SA4503 and [^18^F]Fluspidine, with specific binding in the brain and other S1R-rich organs comparable to the two reference radiotracers. However, it is eliminated too slowly from the brain and shows irreversible brain kinetics [62]. In contrast, compound **3b** has lower brain uptake than compound **3a** but displays faster brain clearance. Blocking studies showed a higher reduction in brain uptake, consistent with a decrease in lipophilicity [63]. Baum et al. [108] compared (*R*)-[^18^F]Fluspidine, (*S*)-[^18^F]Fluspidine, and compounds **3a** and **3b** in NHPs. All four compounds demonstrated high brain uptake. (*S*)-[^18^F]Fluspidine and compound **3b** showed rapid washout, whereas (*R*)-[^18^F]Fluspidine and compound **3a** exhibited much slower clearance from the brain. Notably, compound **3b** displayed a more heterogeneous uptake pattern. The distribution profiles of all four compounds were consistent with that of [^11^C]SA4503 in humans. Additionally, (*S*)-[^18^F]Fluspidine and compound **3b** were compared to [^11^C]SA4503 in monkeys. Both showed high uptake and a distribution similar to [^11^C]SA4503, although the latter displayed a slower washout. Moreover, [^11^C]SA4503 exhibited higher nonspecific binding than (*S*)-[^18^F]Fluspidine and compound **3b**, possibly due to its lower S1R affinity [108]. Compounds **4a** and **4b** exhibited good brain uptake despite their low logD_7.4_ values. Among the two, compound **4b** showed superior initial brain uptake and greater specific binding [64,65]. Regarding [^18^F]FTC-146, studies in mouse models showed that it rapidly crosses the BBB, reaches peak brain uptake within the first few minutes, and then slowly washes out. This kinetic profile is similar to that of [^18^F]FM-SA4503 and [^18^F]Fluspidine. Pre-treatment with haloperidol or the non-labeled reference led to a marked reduction in [^18^F]FTC-146 binding in the brain—approximately 80% [76]. [^18^F]FTC-146 was also evaluated in rats and squirrel monkeys, where it demonstrated widespread uptake and distribution throughout the brain, showing a similar behavior to [^11^C]SA4503 and [^18^F]FE-SA4503. In squirrel monkeys, high specific binding was confirmed through haloperidol blockade studies; however, accumulation of radioactivity in the skull was also observed, which could pose a problem, as additional radioactivity in the skull may affect PET analysis. However, subsequent studies conducted in humans found no evidence of accumulation into the skull [109]. Regarding [^18^F]FPS and [^18^F]SFE, in the rat brain model, both exhibited the highest average radioactivity uptake in the posterior and frontal cortices, with slightly lower uptake observed across all other examined regions, including the striatum, cerebellum, medulla pons, midbrain, hippocampus, and hypothalamus. A key difference between the two compounds lay in their pharmacokinetics: [^18^F]SFE reached its peak uptake at approximately 5 min p.i. and subsequently showed progressive clearance across all brain regions, whereas [^18^F]FPS demonstrated no observable decrease in radiotracer concentration within the same timeframe. This clearance behavior was considered an advantage of [^18^F]SFE over [^18^F]FPS. Both tracers exhibited high specific binding [78,110]. When comparing [^18^F]SFE to [^18^F]FE-SA4503 in the mouse brain, both showed similar uptake levels; however, [^18^F]FE-SA4503 demonstrated slower clearance. Additionally, [^18^F]FE-SA4503 displayed slightly higher non-specific binding than [^18^F]SFE [111]. [^18^F]TZ3108, evaluated in the rat model, showed uptake patterns consistent with the distribution of S1R in the body, with pronounced accumulation in the lungs and brain. An increase in liver uptake was also observed during imaging, likely indicating hepatobiliary clearance. In NHPs, [^18^F]TZ3108 readily crossed the BBB and demonstrated high brain uptake with a regional distribution similar to that in rats, enabling excellent anatomical visualization. However, its brain kinetics were relatively slow, reaching a peak at around 45 min and maintaining stable concentrations for over 120 min. Blocking studies with cold TZ3108 or Yun-122 confirmed specific binding [100]. Additionally, both enantiomers, (−)-[^18^F]TZ3108 and (+)-[^18^F]TZ3108, have been investigated in rats and NHPs. Despite its lower S1R affinity, the (−)-enantiomer showed faster brain clearance compared to the racemate, reaching its peak at 30 min versus 45 min. Therefore, (–)-[^18^F]TZ3108 is considered superior to both the racemic mixture and the eutomer alone in terms of pharmacokinetics. In blocking studies, (−)-[^18^F]TZ3108 reduced uptake in most brain regions [74]. Among the derivatives of TZ3108, only compounds **5b** and **6b** were studied in vivo in mice. Compound **5b** entered the CNS with a maximum Standardized Uptake Value (SUV) of 2.1 at 5 min p.i., followed by a progressive decrease from 0 to 60 min, whereas compound **6b** reached a higher maximum SUV of 4.1. Blocking studies with haloperidol and TZ3108 reduced brain uptake by 5–10% and 30–35%, respectively, confirming specific binding. In macaques, all six radiolabeled compounds were evaluated and successfully entered the brain, displaying distinct uptake and washout profiles. Among them, compound **7c** showed the highest brain uptake, with an SUV greater than 2.0 at 5 min p.i. Compounds **6b** and **6c**, however, failed to reach equilibrium during a 2 h scan, making them the least suitable candidates for further clinical development. In contrast, compounds **5b** and **5c** demonstrated good brain penetration, favorable washout kinetics, and specific uptake in brain regions with high S1R density [75]. In the mouse model, both [^11^C]HCCO923 and [^11^C]HCCO929 demonstrated similar brain distribution patterns and exhibited specific and selective binding to S1R. Between the two compounds, [^11^C]HCCO929 displayed superior kinetic properties and higher specific binding [70]. [^18^F]CNY-05, in brain imaging studies, successfully crossed the BBB and showed rapid brain uptake with a favorable cerebral clearance profile. However, the blocking studies using SA4503 revealed an unexpected increase in radioactivity levels. In NHPs, the SUV stabilized within the first minutes p.i. and peaked at approximately 40 min. Tracer washout exhibited promising potential for clinical translation. Additionally, [^18^F]CNY-05 was evaluated in 5xFAD transgenic mice, a model for AD. The tracer uptake in all brain regions of AD mice was significantly lower than that observed in wild-type (WT) controls. Notably, the hypothalamus showed markedly reduced radioactivity accumulation in AD mice. These findings support the potential application of [^18^F]CNY-05 in the imaging of AD-related pathological changes [72]. Compound **9**, initially designed for the investigation of D4 receptors, exhibited high brain uptake and a high degree of specific binding in a monkey model, as confirmed by blocking studies [84]. As for vesamicol derivatives, [^11^C]-(+)-pMV shows a regional distribution pattern in the rat brain similar to [^11^C]SA4503, but with lower specific binding [66].

The radioiodine compound [^124^I]IPAG was investigated in an LNCaP xenograft model with regard to possible tumor imaging. It showed specific uptake; however, after a period of 4 h, the activity became non-specifically distributed. Tumor visualization became evident after 24 h, as radioactivity was cleared from non-target organs with minimal S1R expression, similarly to observations for **5** [80]. [^11^C]PB183 and [^11^C]PB212 are unable to cross the BBB and were therefore investigated for imaging prostate cancer or assessing peripheral S1R expression, respectively. In blocking studies using haloperidol, Fluspidine, and SA4503, [^11^C]PB212 demonstrated specific and reversible binding in the spleen. Moreover, [^11^C]PB212 is an S1R antagonist, with the potential to provide additional information about S1Rs compared to the agonists, which are more represented among the S1R PET radioligands [68,69]. To the best of our knowledge, however, this aspect has not been investigated further. Compound **8** was intended to provide a dual S1/S2R radiotracer, able to image prostate cancer. In mice bearing PC-3 tumors, increasing tumor accumulation was shown, reaching a peak at 1.5 h, along with rapid renal and hepatobiliary excretion. Blocking studies with haloperidol demonstrated a reduced accumulation in the tumor, indicating specific binding [83].

## 5. In Vitro and Ex Vivo Autoradiographic Studies

In vivo biodistribution and blocking studies are key components of PET tracer validation, providing essential data on pharmacokinetics, uptake, target specificity, and off-target binding. These evaluations are critical to confirm tracer suitability before clinical translation [103]. Characterization of the radiotracers also in pathological models (preclinical models of cancers and neurodegenerative diseases that involve S1R) is also performed for the most promising radioligands, in the perspective of further validation towards the translation into the clinic.

Many of the compounds discussed in this review have been investigated using in vivo assays but have not been evaluated through in vitro or ex vivo autoradiography (ARG). Although modern dedicated micro-PET scanners achieve spatial resolutions of approximately 1–2 mm and excellent system sensitivity (enabling precise quantification of radiotracer uptake in small brain structures and longitudinal, dynamic studies within the same animal), ARG remains of extreme importance for validating PET findings by providing high-resolution spatial maps of tracer uptake, confirming binding specificity, and revealing microregional distribution patterns that surpass the resolution limits of in vivo imaging [112,113,114,115,116]. In addition, ARG aligns with the principles of the 3Rs (reduce, refine, replace) in animal research [115]. For both [^11^C]SA4503 and [^18^F]FE-SA4503, ex vivo ARG in rats confirmed the in vivo biodistribution and revealed highly comparable regional uptake patterns. Notably, high tracer accumulation was observed in the vestibular nucleus, temporal cortex, cingulate cortex, inferior colliculus, thalamus, and frontal cortex. Moderate uptake levels were detected in the parietal cortex and caudate putamen, while the cerebellum and hippocampus exhibited relatively low tracer accumulation [47,49]. Compounds **2** and [^18^F]FBFP, structurally related to [^11^C]SA4503, demonstrated a brain distribution pattern consistent with regions known to exhibit high S1R expression, although some differences were observed compared to [^11^C]SA4503. Blocking studies confirmed its specific binding to S1R [53,55]. For [^18^F]FBFP, ex vivo ARG was also employed to explore its potential application in AD by comparing senescence-accelerated prone (SAMP8) mice with senescence-accelerated resistant (SAMR1) mice. Consistent with the ex vivo autoradiography results observed in rats, a high level of [^18^F]FBFP accumulation was detected in brain regions with known high S1R expression in SAMR1 mice. In contrast, the same regions showed a reduced accumulation of the radiotracer in age-matched SAMP8 mice [53]. For [^18^F]IAM6067, in vitro autoradiography in rats showed strong concordance with in vivo PET imaging, further validating its specificity and selectivity as an S1R radiotracer [107]. Regarding the spirocyclic compounds, both [^18^F]WMS-1813 and [^18^F]Fluspidine show an ex vivo ARG pattern consistent with in vivo distribution in brain regions known to be rich in S1R, particularly in the facial nucleus. For [^18^F]Fluspidine, the lowest concentration of binding sites was observed in the anterior part of the olfactory bulb [57,96]. Compound **3a** displayed a regional brain distribution similar to that of [^18^F]Fluspidine; however, unlike [^18^F]Fluspidine, only moderate accumulation in the facial nucleus was detected [62]. Compound **3b**, in ex vivo ARG analysis, displayed high uptake in brain areas rich in S1R, which is consistent with the brain distribution of [^11^C]SA4503. Similarly to [^18^F]Fluspidine, it has shown low binding site concentration in the anterior part of the olfactory bulb [63]. For both compounds **4a** and **4b**, ex vivo autoradiography confirmed the in vivo biodistribution in brain regions with high concentrations of S1R. Blocking studies with haloperidol or SA4503 further supported the specificity of binding [64,65]. As for [^18^F]FTC-146, administration of the compound resulted in an accumulation in the midbrain, facial nucleus, cortex, and hippocampus. Uptake was also observed in the cerebellum, although to a lesser extent than in the midbrain and cortex, whereas the corpus callosum lacked [^18^F]FTC-146 binding. Blocking studies demonstrated a marked reduction in tracer uptake throughout the mouse brain. Overall, ex vivo autoradiography confirmed the in vivo biodistribution in both mice and rats [76,109]. For [^18^F]CNY-05, in vitro autoradiography confirmed its S1R-specific binding. Similarly to the in vivo studies, in vitro ARG performed in the 5xFAD mouse model revealed a heterogeneous distribution of radioactivity across brain regions and a marked reduction in brain uptake in 5xFAD mice compared to WT controls [72]. The regional distribution of (+)-[^11^C]pMV in the brain was very similar to that of [^11^C]SA4503 [66]. For [^11^C]PB212, in vitro autoradiography was performed using brain tissues from both WT and S1R KO mice. Specific binding was demonstrated through blocking studies using haloperidol, SA4503, and Fluspidine [69]. For compound **9**, in vitro autoradiography was performed to evaluate its binding affinity toward both the D_4_ receptor and the S1R. The observed distribution did not correspond to the known brain distribution of the D_4_ receptor. Blocking studies with (+)-pentazocine conclusively demonstrated specific binding to S1R [84].

## 6. In Vitro and In Vivo Metabolism

The chemical identification of radiometabolites serves two primary purposes: first, to accurately quantify the proportion of unchanged (parent) radiotracer present in the plasma; second, to identify and correct for any unintended accumulation of radiometabolites in non-target tissues. The formation of radiometabolites can pose significant challenges in PET imaging, as these byproducts often exhibit markedly different pharmacokinetics and biodistribution compared to the original tracer [117]. In the case of brain imaging agents, it is preferable that metabolic breakdown occur outside the CNS, resulting in more polar, less lipophilic compounds that are unlikely to cross the BBB or interfere with the specific binding to the target protein [118]. Regarding [^11^C]SA4503, its metabolic profile varies significantly across species. In rats, the radiotracer exhibited slow peripheral metabolism, with more than 99% of the intact compound present in the frontal cortex and 77% remaining in plasma 30 min post-injection (p.i.), and no detectable ^11^C-labeled metabolites in the brain [47]. In contrast, cats showed rapid metabolism: the fraction of unchanged [^11^C]SA4503 in plasma declined sharply to 21.9% at 30 min, 12.5% at 60 min, and 11.0% at 90 min after administration [119]. In monkeys, the radiotracer underwent intermediate metabolic degradation, with the parent compound accounting for 57%, 42%, and 31% of plasma radioactivity at 30, 60, and 90 min, respectively, under baseline conditions. Under carrier-added conditions, these values slightly decreased to 43%, 32%, and 29% at the corresponding time points. These findings indicate that [^11^C]SA4503 undergoes peripheral metabolism more rapidly in monkeys than in rats but less rapidly than in cats [105]. As for [^18^F]FE-SA4503, at 30 min p.i., the percentage of unchanged radiotracer in plasma was 49 ± 9.5% in mice and 55 ± 24% in monkeys. In mice, a small amount of metabolites was detected in brain tissue (the percentage of unchanged radiotracer was 93.4 ± 2.6%), and defluorination was observed. In contrast, in monkeys, neither brain metabolites nor defluorination were observed [49,106]. Instead, the percentage of unchanged [^18^F]FM-SA4503 in mouse plasma was only 8.4 ± 1.2% at 60 min p.i. In brain tissue, only a small fraction of metabolites was detected, with 94.1 ± 1.0% of the radiotracer remaining unchanged [51]. Regarding the [^11^C]SA4503 derivatives, radiotracers **1a** and **1b**, both containing an amide group, showed parent compound percentages of 33.7% and 75.4%, respectively, in mouse brain at 30 min p.i. In plasma, the intact forms of **1a** and **1b** accounted for 10.1% and 46.9%, respectively, at the same time point. In the liver, the corresponding values were 3.3% for **1a** and 49.3% for **1b**. For radiotracer **1c**, the parent compound represented 10.6%, 92.4%, and 68.3% of the total radioactivity in mice plasma, brain, and liver, respectively, at 30 min p.i. [52]. In contrast, rapid metabolism in mice was detected for [^18^F]IAM6067, with only 10.1 ± 8.6% of the intact tracer remaining in plasma at 10 min p.i. However, over 91% of the parent compound was retained in the brain at 10, 20, and 60 min p.i. Two more hydrophilic metabolites were identified as products of [^18^F]IAM6067 metabolism [107]. For racemic [^18^F]FBFP and its *R*- and *S*-enantiomers, approximately 95% of the total radioactivity in the mouse brain at 60 min p.i. was attributable to the parent compound [55,85].

Regarding spirocyclic compounds in mice at 60 min p.i., the percentage of unmetabolized [^18^F]WMS-1813 and [^18^F]Fluspidine in plasma was over 85% and approximately 50%, respectively. In the brain, the parent compound represented over 95% and ~98% of total radioactivity, respectively. For [^18^F]Fluspidine, 57% of the intact tracer was detected in liver homogenates of mice at 60 min, along with two main hydrophilic metabolites. For both radiotracers, no radiometabolites were found in the brain; however, unbound [^18^F]Fluoride was detected in plasma. The findings indicate that [^18^F]Fluspidine undergoes a metabolic process similar to [^18^F]WMS-1813, but at a faster rate [57,96]. The metabolism of the *R*- and *S*-enantiomers of both compounds was also investigated in vitro using rat liver microsomes. In both cases, the (*R*)-enantiomer was metabolized more rapidly than the (*S*)-enantiomer. For [^18^F]WMS-1813, only 28% of the (*R*)-form remained after 30 min of incubation, compared to 54% of the (*S*)-form [60]. For [^18^F]Fluspidine, ~72% of both enantiomers remained unchanged after 30 min; however, after 90 min, the (*S*)-enantiomer showed significantly greater stability, with 58% remaining intact versus 33% for the (*R*)-counterpart [61]. The stereospecificity of the metabolism of [^18^F]Fluspidine was confirmed in a pig model, where at 16 min p.i., 23% of the (*R*)-enantiomer and 45% of the (*S*)-enantiomer remained unmetabolized [120]. In Rhesus monkeys, 60 min p.i., plasma levels of unmetabolized tracer were 37% for the (*R*)-enantiomer and 35% for the (*S*)-enantiomer [108]. In human plasma, 91% of the (*S*)-enantiomer remained intact at 30 min p.i. It was shown that the primary metabolic pathway involves hydroxylation of the fluoroethyl chain followed by glucuronide conjugation. Furthermore, debenzylation has been identified as a secondary metabolic route [121]. For the Fluspdine derivative **3a** investigated in male ICR mice, the percentage of parent tracer in the liver was 40% at 30 min p.i., and no radiometabolites were detected in the brain [62]. For compound **3b**, investigated under similar conditions, the percentages of unchanged radiotracer were 77% in plasma, 49% in the liver, and 93% in the brain [63]. In monkeys, the parent fraction at 60 min p.i. was 18% for compound **3a** and 19% for compound **3b**, indicating a faster metabolic depletion in comparison to both the enantiomers of [^18^F]Fluspidine [108]. Compound **4a** exhibited very rapid metabolism in mice, with only 6% of the unchanged parent compound remaining 60 min p.i., likely due to its ketal as a vulnerable function [64]. Indeed, compound **4b**, which lacks the ketal group, showed a slower, though still relatively fast, metabolic degradation. At 15 min after radiotracer injection, 13% of the total radioactivity was detected in plasma samples and 84% in brain homogenates of ICR mice, respectively [65]. Regarding [^18^F]FTC-146, in mouse models, the percentages of intact parent compound in the liver and plasma were 34% and 60% at 30 min, respectively, and 27% and 50% at 60 min, respectively. The percentage of intact tracer in the brain remained at 100% throughout the study duration [76]. In rat models, metabolism was faster than in mice, with 25.3% and 19.5% of the parent compound detected in plasma at 30 and 60 min, respectively. In monkeys, the metabolic profile was comparable to that observed in mice. Similarly to mice, 100% of the parent compound was observed in the brain at all time points analyzed both in mice and monkeys [109]. In humans, 43% and 23% of peak radioactivity remained in circulation at 30 and 120 min p.i., respectively. As seen in preclinical studies, only intact [^18^F]FTC-146 was detected in the rat brain at 15, 30, and 60 min p.i. [122]. Metabolism studies of [^18^F]TZ3108 were conducted in both rats and NHPs, revealing similar results across species. The percentages of intact parent compound in plasma of rats and NHPs were 88.5% and 97.9% at 5 min, 86.5% and >86% at 30 min, and 78.2% and ~70% at 60 min, respectively. The major metabolite identified in both species does not cross the BBB [100]. As for vesamicol derivatives, the percentage of unchanged [^11^C]-(+)-pMV in the brain was 95%, while in plasma at 30 min p.i., 21% was detected [66]. Regarding [^18^F]CNY-05, in NHPs, more than 50% of the parent compound remained in plasma at 60 min p.i. No radiometabolites were found to cross the BBB [72]. For compound **8**, analysis of radioactivity in tumor and blood samples at 5 min p.i. showed that more than 99% of the extracted radioactivity corresponded to the intact compound. However, by 30 min p.i., the proportions had decreased to 52% in the tumor and 24% in the blood. In contrast, in the liver, intact compound **8** accounted for 46% and 67% of total radioactivity at 5 and 30 min p.i., respectively [83]. Regarding compound **2**, metabolite analysis in monkey plasma indicated that it was considerably stable, with approximately 80% of the total radioactivity corresponding to the intact parent compound even at 90 min p.i. [84].

## 7. Kinetic Characterization of PET Radiotracers

Kinetic studies are fundamental in the development of novel PET radiotracers because they provide a time-resolved, quantitative characterization of tracer behavior in vivo, encompassing delivery, target engagement, metabolism, and clearance. By acquiring dynamic PET data immediately after tracer injection and applying compartmental models, each compartment representing a distinct physiological or biochemical state, researchers can estimate rate constants (k_1_, k_2_, k_3_, k_4_) that reflect key processes such as blood–tissue transfer, receptor binding, intracellular trapping, and dissociation [123]. Such analyses not only yield biologically meaningful parameters beyond static SUVs but also guide rational tracer design (e.g., optimizing affinity and lipophilicity), inform selection of optimal imaging time points, and underpin protocol simplification strategies for clinical translation [124]. In this framework, Logan graphical analysis provides a model-independent, linear regression approach for reversible tracer kinetics, enabling the estimation of total distribution volume (V_t_) or distribution volume ratio (DVR) from dynamic PET data once a pseudo-equilibrium between tissue and plasma has been achieved [125]. By incorporating Logan analysis into the kinetic evaluation of novel PET tracers, researchers can efficiently screen candidates for favorable reversible binding profiles, optimize scanning protocols, and accelerate the transition of promising compounds toward clinical application [126]. For [^11^C]SA4503, a three-compartment, two-tissue kinetic model was employed to estimate parameters including k_1_, k_2_, k_3_, k_4_, and blood–brain delay. In addition, a Logan graphical analysis was applied to assess reversible binding. The brain uptake of [^11^C]SA4503 exhibited a biphasic pattern, with a rapid initial uptake followed by a slow, progressive accumulation over time. Notably, [^11^C]SA4503 demonstrated prolonged retention in the brain, consistent with its target-binding profile [127,128]. For both [^11^C]SA4503 and [^18^F]FE-SA4503, a shortened acquisition protocol of 60 min has been shown to provide sufficient kinetic information. In contrast, [^18^F]FE-SA5845 requires a longer acquisition time of up to 2 h to achieve reliable kinetic modeling results [106,129]. For [^18^F]WMS-1813, [^18^F]Fluspidine and [^18^F]TZ3108, kinetic studies were fundamental to determining which enantiomer was more suitable. For both [^18^F]WMS-1813 and [^18^F]Fluspidine, the (*S*)-enantiomer showed more favorable kinetics [60,61]. Both enantiomers of Fluspidine were evaluated in porcine brain using a two-compartment tissue model to determine binding potential (BP_ND_) and V_T_, with comparisons made to [^11^C]SA4503. The (*S*)-enantiomer displayed lower BP_ND_ and V_T_ values than both [^11^C]SA4503 and the (*R*)-enantiomer. Additionally, B_max_ values, which represent the maximum amount of radioligand that can bind specifically to receptors when all available binding sites are fully occupied, for the (*R*)-enantiomer were approximately five-fold higher than those of the (*S*)-enantiomer, likely reflecting differences in their respective *K*_i_ values. Notably, the (*S*)-enantiomer exhibited faster equilibration kinetics, including a higher *k*_4_ value, suggesting more rapid dissociation from the target site. Due to these favorable kinetic properties, specifically shorter scan durations and slower peripheral metabolism, the (*S*)-enantiomer shows greater potential for clinical application in brain imaging [120]. In NHPs, binding potential followed the order: compound **3a** > (*R*)-[^18^F]Fluspidine > compound **3b** > (*S*)-[^18^F]Fluspidine, consistent with their *K_i_* values. V_T_ estimates were also time-dependent: reliable measurements were obtained within 90 min for (*S*)-[^18^F]Fluspidine and compound **3b**, while longer acquisitions were required for (*R*)-[^18^F]Fluspidine and compound **3a**. However, due to their very slow kinetics, (*R*)-[^18^F]Fluspidine and compound **3a** are considered unsuitable for clinical translation [108]. [^18^F]CNY-05 was also evaluated in NHPs from a kinetic perspective, revealing a lower V_T_ compared to both enantiomers of [^18^F]Fluspidine as well as compounds **3a** and **3b**. As for [^18^F]FTC-146, the V_T_ was not determined in NHPs, to the best of our knowledge, making it impossible to compare its kinetic profile with that of other radiotracers [72]. Regarding [^18^F]TZ3108, the (−)-enantiomer, although characterized by lower in vitro affinity for the S1R, was shown to exhibit significantly faster clearance kinetics compared to the racemate. Specifically, (−)-[^18^F]TZ3108 reached its peak brain uptake at approximately 30 min p.i., whereas the racemic mixture peaked at around 45 min. Importantly, (−)-[^18^F]TZ3108 demonstrated greater in vivo imaging ability as an S1R tracer than [^11^C]SA4503, while simultaneously offering more favorable pharmacokinetic properties. These include improved washout rates and reduced nonspecific binding, making (−)-[^18^F]TZ3108 a promising candidate for clinical PET imaging of the S1R system [74].

## 8. Dosimetric Evaluation and Clinical Translation

In the context of clinical translation of novel PET radiotracers, preclinical dosimetry and toxicity studies are crucial to rule out safety issues at an early stage so that there are no concerns for first-in-human trials. Dosimetric studies allow for the estimation of radiation absorbed doses in organs and tissues following radiotracer administration, typically using animal models. This step is crucial to ensure that exposure levels are consistent with human safety thresholds, as recommended by the International Commission on Radiological Protection [130]. In parallel, preclinical toxicity studies, usually performed in animal models according to the International Conference on Harmonization (ICH) guidelines, are intended to detect non-radiological adverse effects, such as chemical toxicity of the compound or its metabolites. As for the Effective Dose (ED) limits, most European countries follow a general threshold of 10 mSv, in accordance with ICRP recommendations, while the U.S. Food and Drug Administration (FDA) sets the ED limit at 50 mSv. However, for radiotracers investigated under the authority of a Radioactive Drug Research Committee (RDRC) in the United States, dose constraints are applied both to the whole body and to individual organs. Furthermore, the injected mass must not induce any pharmacologically detectable effect. For most novel ^18^F-labeled PET radiotracers, the injected mass is maintained within the range of 1 to 5 µg per dose [131]. Human radiation dosimetry studies of [^11^C]SA4503 have shown that the highest absorbed doses were observed in the liver, kidneys, and pancreas. However, the overall radiation burden remained within acceptable limits for clinical application [104]. Subsequently, [^11^C]SA4503 was employed together with [^15^O]H_2_O in humans to compare the radioligand’s brain kinetics with the regional cerebral blood flow (rCBF) in the same subject. Results showed that regional variations in binding potential are due to differences in S1R density and are not likely to reflect perfusion-related artifacts. Therefore, [^11^C]SA4503 represents a suitable radiotracer for quantifying S1R expression in the human brain [132]. To date, no other compound derived from [^11^C]SA4503 has been investigated in humans. As for [^18^F]Fluspidine, both the (*R*)- and (*S*)-enantiomers have been investigated in mouse models to determine radiation dosimetry. The (*S*)-[^18^F]Fluspidine enantiomer was further evaluated in four human volunteers, revealing an ED of 21.0 ± 1.3 μSv/MBq, which falls within the acceptable range for human studies. The radiotracer exhibited faster elimination from the brain, liver, stomach, and spleen, while clearance was comparatively slower in the bone marrow. [133]. As with [^11^C]SA4503, no other compounds derived from (*S*)-[^18^F]Fluspidine have been studied in humans. Regarding [^18^F]FTC146, dosimetric analysis indicated the highest radiation exposure in the spleen, urinary bladder, and, most prominently, bone tissue. Nevertheless, preclinical toxicity studies supported its suitability for human use [134]. In a first-in-human study involving 10 healthy volunteers, [^18^F]FTC146 showed an effective dose of approximately 11.0 μSv/MBq. No adverse events were reported, and absorbed doses in critical and radiosensitive organs were considered acceptable and safe [122]. In terms of biodistribution, [^18^F]FTC146 demonstrated a faster brain washout in humans compared to NHPs, though its kinetics remained relatively slow. Clearance was predominantly renal, with elimination via the kidneys and bladder. Unlike in monkeys, no significant skull uptake was observed in humans, but notable thyroid uptake was detected [135]. Other radioligands evaluated in the past through dosimetric tests to assess their potential for clinical translation are [^18^F]FPS and [^18^F]SFE. For both compounds, each organ is estimated to receive approximately 0.012–0.015 mGy/MBq, with the adrenal glands identified as the critical organs. Overall, both tracers appear to be safe for human use, with a maximum administered activity of 185 MBq and a mass dose of less than 2.8 μg for [^18^F]FPS and less than 4 μg for [^18^F]SFE per injection. However, neither compound has been further investigated in human studies [110,136].

## 9. Role of PET Imaging in the Assessment of Pathophysiological Processes

Only the most relevant PET radiotracers have been employed to investigate the role of S1R in pathophysiological processes and receptor occupancy studies. Among them, [^11^C]SA4503 stands out as one of the earliest and most widely used S1R PET tracers, often considered the gold standard in this field. It has been utilized in a variety of applications. Using [^11^C]SA4503, it was demonstrated that fluvoxamine and donepezil bind to S1R in the human brain at therapeutic doses following a single oral administration [137,138]. The tracer was also used in receptor occupancy studies in rat brains, where pretreatment with donepezil was shown to increase [^11^C]SA4503 metabolism in a dose-dependent manner [139]. PET imaging with [^11^C]SA4503 has revealed that the iris-ciliary body and retina are rich in S1R expression [140]. Additionally, the tracer has been employed in preclinical in vivo drug screening to identify novel S1R agonists [141] and to detect spontaneous pituitary tumors in aged rats, distinguishing them from normal pituitary tissue and brain tumors [119]. Furthermore, [^11^C]SA4503 has also been demonstrated to be suitable to quantify S1R binding of unlabeled ligands in competitive binding studies [142]. (*S*)-[^18^F]Fluspidine has been investigated in patients with acute major depressive disorder, and an increased S1R binding in specific brain areas was found, which possibly reflects different depression subtypes. Furthermore, the authors assumed that this, together with a correlation between severity and receptor density, gives an indication for neuroadaptive counter regulation in such disease states [143]. The radioligand has also been used in glioblastoma studies in an orthotopic mouse model, revealing the presence of S1R in the infiltrative tumor margin, highlighting its potential applicability in tumor imaging as well [144]. [^18^F]Fluspidine was also used in healthy volunteers and in patients with HD to demonstrate that pridopidine acts as a selective ligand for S1R, showing nearly complete S1R occupancy (~90%) and minimal occupancy of S2R/D3R (~3%) [145]. [^18^F]FTC146 has proven to be a valuable tool in the investigation of pain-related conditions. It has been successfully employed to visualize nerve damage in a rat model of neuropathic pain, enabling non-invasive detection of affected areas. This highlights the potential of [^18^F]FTC146, in PET/MRI combination, for the study of neuropathic pain [146]. In a clinical context, [^18^F]FTC146 was used in a pilot study involving five healthy volunteers and five patients with chronic pelvic pain, demonstrating its possible utility as a diagnostic tool in such conditions [147]. Furthermore, [^18^F]FTC146 has been applied in receptor occupancy studies of blarcamesine in rat models of Fragile X syndrome, where it revealed that blarcamesine binds to S1R in a dose-dependent manner [148]. As for [^18^F]FPS, it has been used to demonstrate that neuroactive steroids can occupy the S1R in the brain of mice [149]. [^18^F]TZ3108 has shown potential in detecting changes in the quantity and density of S1Rs in the liver of subjects with metabolic-associated fatty liver disease (MAFLD). Specifically, it enables the assessment of liver function impairment during the early stages of MAFLD, thereby overcoming the limitations associated with the use of [^18^F]FDG. As such, [^18^F]TZ3108 may serve as a promising tool for the early diagnosis of MAFLD [150,151].

## 10. Conclusions

PET radiotracers hold enormous potential in contributing to the elucidation of receptors’ physio-pathological roles, helping in the pathways that lead to their exploitation as therapeutic and diagnostic targets. The growing interest in the S1 pluripotent chaperone as a promising druggable target has amplified the need for reliable imaging agents to be exploited as diagnostic tools or as valuable means to assist in the drug-development process. In this review, we provide a comprehensive analysis of the most important PET radiotracers developed for the S1R since the early 2000s, comparing them across all key parameters relevant to radiotracer evaluation. The different classes of S1R radiotracers were described with a focus on the specific structural modifications that have influenced the S1R affinity and selectivity. A comparative assessment of their in vivo performance across different animal models and in vitro autoradiography data was conducted together with an analysis of the differences in metabolism and pharmacokinetics. This overview culminates in the evaluation of the dosimetric profiles and the potential for clinical translation of these compounds. Such a comprehensive overview highlights the important efforts to be devoted towards ideal PET radiotracers in which an optimal balance between binding affinity and kinetic profiles needs to be reached. The most notable radiotracers include [^11^C]SA4503, one of the earliest S1R PET ligands developed; [^18^F]-(*S*)-Fluspidine; and [^18^F]FTC-146, which has undergone and is still undergoing clinical evaluations as a diagnostic for diverse diseases (e.g., chronic pain [147], sciatica [152], and osteosarcoma [153]). Importantly, these radiotracers have also served for the characterization of novel S1R based therapeutics through receptor occupancy studies that have revealed the S1R-specific interaction of these compounds, thus supporting their S1R-mediated effect (e.g., Blarcamesine and Pridopidine). Analogously, these radiotracers have served to clarify the interactome of already known drugs whose mechanisms of action have been revealed to also be S1R-mediated (e.g., Fluvoxamine and Donepezil). Among the more recent developments, there are some S1R radiotracers looking promising as diagnostic agents, such as [^18^F]TZ3108 and [^18^F]CNY-05 (the former for the study of MAFLD and the latter CNY-05 for exploring the role of S1R in neurodegenerative diseases), but their potential for clinical use remains to be seen in the coming years. While studies with the current S1R leading radiotracers are still ongoing, the plethora of S1R PET agents will continue to expand in the pursuit of an optimal S1R imaging tool that overcomes the limitations of existing radiotracers and fully enables the exploitation of S1R in health and disease.

## Figures and Tables

**Table 2:** S1R radiotracers labeled with ^11^C: radiochemical routes and corresponding RCYs.

Radioligand	Radiochemical Route	Radiochemical Yield	References
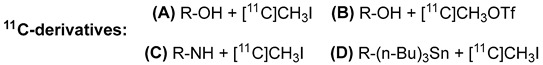			
[^11^C]SA4503	A	21–31%	[47]
[^11^C]PB-212	A	16–33%	[69]
**(**−**)-5b**	B	20%	[75]
**(+)-5c**	B	18%	[75]
**(**−**)-6b**	B	20%	[75]
**(+)-6c**	B	18%	[75]
**(**−**)-7b**	B	17%	[75]
**(+)-7c**	B	16%	[75]
[^11^C]HCCO923	A	6–15%	[70]
[^11^C]HCCO929	C	3–8%	[70]
[^11^C]-(+)-pMV	D	8–19%	[66]
**9**	A	32–59%	[84]

**Table 3:** S1R radiotracers labeled with ^18^F: radiochemical routes and corresponding RCYs.

Radioligand	Radiochemical Route	Radiochemical Yield	References
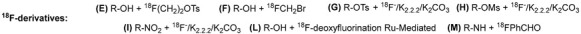			
[^18^F]FE-SA4503	E	4–7%	[49]
[^18^F]FM-SA4503	F	3–17%	[51]
[^18^F]WMS-1813	G	35–48%	[57]
[^18^F]Fluspidine	G	from 35–45% to 37 ± 8%	[96,97]
**3a**	G	35–60%	[62]
**3b**	E	8–10%	[63]
[^18^F]FTC-146	G	from 4–7% to 41.7 ± 4.4%	[76,98]
[^18^F]FPS	H	56–70%	[78]
[^18^F]SFE	H	59 ± 8%	[79]
[^18^F]TZ3108	I	18–24%	[100]
[^18^F]CNY-05	L	25–40%	[72]
**4a**	E	15%	[64]
**4b**	G	12–35%	[65]
[^18^F]FBFP	M	20–30%	[55]
**8**	E	35–50%	[83]
[^18^F]IAM6067	G	18%	[54]
**1a-c**	G	42–55%	[52]
**2**	G	30–50%	[53]

**Table 4:** S1R radiotracer labeled with ^124^I: radiochemical route and corresponding RCY.

Radioligand	Radiochemical Route	Radiochemical Yield	Reference
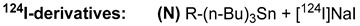			
[^124^I]IPAG	N	100%	[80]

## Data Availability

No new data were created or analyzed in this study. Data sharing is not applicable.

## Abbreviations

SRs, sigma receptors; S1R, sigma-1 receptor; S2R, sigma-2 receptor; ER, endoplasmic reticulum; MAMs, mitochondrion-associated membranes; Binding Immunoglobulin Protein, BiP; IP_3_R, 1,4,5-trisphosphate receptor; CNS, central nervous system; PD, Parkinson’s disease; AD, Alzheimer’s disease; HD, Huntington’s disease; PET, positron emission tomography; CT, computed tomography; MRI, magnetic resonance imaging; ^11^C, carbon-11; ^18^F, fluorine-18; SAR, structure–activity relationship; RCYs, radiochemicalyields; DMF, DMSO, dimethyl sulfoxide; A_m_, dimethylformamide, molar activity; TFA, trifluoroacetic acid; SN_Ar_, aromatic nucleophilic substitution; [^18^F]FETos, 2-[^18^F]fluoroethyl tosylate; NHPs, nonhuman primates; SUV, Standardized Uptake Value; ARG, ex vivo autoradiography; SAMP8, senescence-accelerated prone; SAMR1, senescence-accelerated resistant; p.i., post-injection; V_t_, total distribution volume; DVR, distribution volume ratio; BPND, binding potential; ICH, International Conference on Harmonization; ED, Effective Dose; FDA, Food and Drug Administration; RDRC, Radioactive Drug Research Committee; rCBF, regional cerebral blood flow; MAFLD, metabolic-associated fatty liver disease.
